# Microalgae as Sustainable Biofactories to Produce High-Value Lipids: Biodiversity, Exploitation, and Biotechnological Applications

**DOI:** 10.3390/md19100573

**Published:** 2021-10-14

**Authors:** Tomásia Fernandes, Nereida Cordeiro

**Affiliations:** 1 Laboratory of Bioanalysis, Biomaterials, and Biotechnology (LB3), Faculty of Exact Sciences and Engineering, University of Madeira, Campus Universitário da Penteada, 9020-105 Funchal, Portugal; tomasia.fernandes@uma.pt; 2 Interdisciplinary Centre of Marine and Environmental Research (CIIMAR), University of Porto, 4450-208 Matosinhos, Portugal

**Keywords:** sustainability, industrial valorization, carbon dioxide fixation, biological activities, polyunsaturated fatty acids, phytosterol, carotenoids

## Abstract

Microalgae are often called “sustainable biofactories” due to their dual potential to mitigate atmospheric carbon dioxide and produce a great diversity of high-value compounds. Nevertheless, the successful exploitation of microalgae as biofactories for industrial scale is dependent on choosing the right microalga and optimum growth conditions. Due to the rich biodiversity of microalgae, a screening pipeline should be developed to perform microalgal strain selection exploring their growth, robustness, and metabolite production. Current prospects in microalgal biotechnology are turning their focus to high-value lipids for pharmaceutic, nutraceutic, and cosmetic products. Within microalgal lipid fraction, polyunsaturated fatty acids and carotenoids are broadly recognized for their vital functions in human organisms. Microalgal-derived phytosterols are still an underexploited lipid resource despite presenting promising biological activities, including neuroprotective, anti-inflammatory, anti-cancer, neuromodulatory, immunomodulatory, and apoptosis inductive effects. To modulate microalgal biochemical composition, according to the intended field of application, it is important to know the contribution of each cultivation factor, or their combined effects, for the wanted product accumulation. Microalgae have a vital role to play in future low-carbon economy. Since microalgal biodiesel is still costly, it is desirable to explore the potential of oleaginous species for its high-value lipids which present great global market prospects.

## 1. Introduction

**Figure 1:**
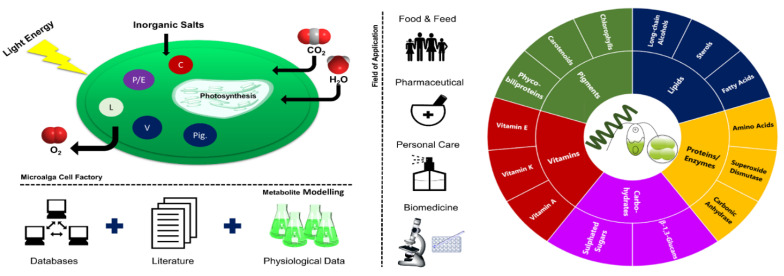
Uncovering the potential of a microalga as a biofactory. C—carbohydrates; P/E—protein/enzymes; L—lipids; V—vitamins; Pig.—pigments.

Microalgae use light energy and inorganic nutrients to produce oxygen and biomass rich in a diversity of value-added compounds (Figure 1) [1]. They can thrive in almost all environments and can be found in oceans, brackish water, freshwater, rocks, and soils [2]. Microalgal biotechnological historical data go back to the Aztec population, who harvested *Arthrospira* from lake Texcoco for food purposes [2,3]. *Arthrospira* has also been collected by local people surrounding Lake Chad and consumed as a nutritional supplement called “dihe” [2]. *Nostoc* species have been used by the Chinese as a food delicacy and for their properties for hundreds of years [2].

**Figure 2:**
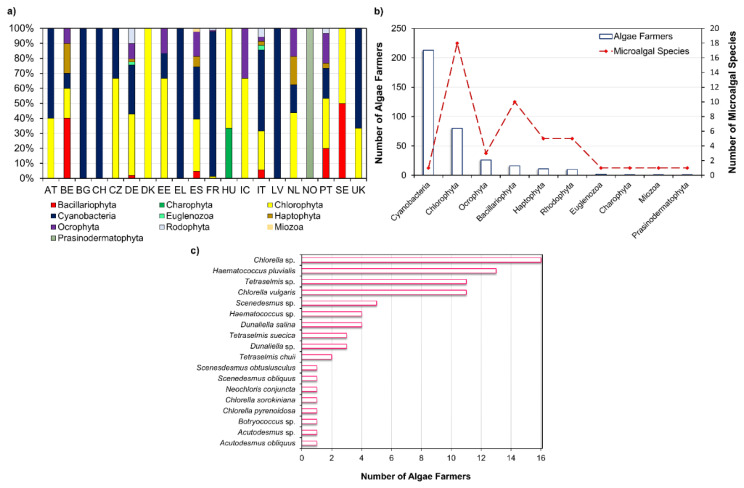
Microalgae production in Europe: (**a**) relative abundance of microalgae at phylum level produced by algae farmers (AT—Austria; BE—Belgium; BG—Bulgaria; CH—Switzerland; CZ—Czech Republic; DE—Germany; DK—Denmark; EE—Estonia; EL—Greece; ES—Spain; FO—Faroe Islands; FR—France; GR—Greenland; HU—Hungary; IC—Iceland; IE—Ireland; IT—Italy; LV—Latvia; NL—the Netherlands; NO—Norway; PT—Portugal; SE—Sweden; UK—the United Kingdom). (**b**) Number of algae farmers against main microalgae phyla and diversity of species exploited. (**c**) Diversity of Chlorophyta species produced by algae farmers. Based on EMODnet database [6].

*Arthrospira platensis*, *Aphanizomenon flosaquae* var. flosaquae, *Chlorella luteoviridis, Chlorella pyrenoidosa*, *Chlorella vulgaris*, and *Auxenochlorella protothecoides* have been on the market as a food or food ingredient and consumed to a significant degree before 15 May 1997 in the European Union market; thus, its access to the market is not subject to the Novel Food Regulation (EU) 2015/2283 [4]. Dried *Tetraselmis chuii*, *Odontella aurita*, and astaxanthin-rich oleoresin from *Haematococcus pluvialis* are microalgal products approved as a novel food and, as the microalgae listed previously, are within the list of microalgae that can be commercialized in the EU [5]. Through Figure 2a, it is possible to visualize that in most European countries, algae farmers mainly produce microalgal species belonging to Cyanobacteria and Chlorophyta phyla, except for Belgium (BE), Norway (NO), and Sweden (SE). This is consistent with the microalgae approved for human consumption which belong to Chlorophyta and Cyanophyta phyla, except for *Odontella aurita*, which belongs to Bacillariophyta phylum.

**Figure 3:**
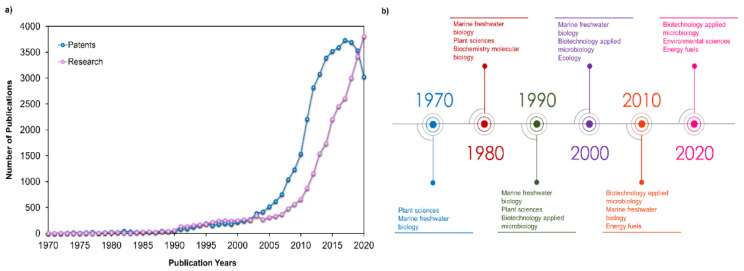
Patenting and research activity in the microalgal field: (**a**) numbers of microalgae-related patents and research publications against publication years; (**b**) timeline with the main research activities categories according to Web of Science. The information used to construct these plots using “microalgae” as topic can be found in Espacenet [9] and Web of Science databases [10].

Health, energy, and human nutrition are the three main applications for microalgal products [7]. However, the energy production from microalgae is experiencing a slow growth compared to other segments [7]. Through Figure 3a, it is possible to observe that both patents and research activities presented an exponential increase between 2004 and 2014. This trend continued for research activities, in contrast to patent publications. These waves of microalgae-related research and development activities are mainly associated with energy-driven trends, and having as the driving force the high crude oil price [8]. After 2015, this driving force was lost due to the development and popularization of electric cars [8]. Figure 3b shows that research activities were mainly focused on the biological sciences before 1990s. From this decade forward, biotechnology-applied microbiology appeared in the main research categories meeting the slight increase in patent and research publications.

Through Figure 2b, it is possible to visualize that the divisions comprising the greatest diversity of microalgal species industrially exploited are Chlorophyta (18) and Bacillariophyta (10) phyla. According to Griffiths et al. [11] most microalgal species considered for biofuel production are either Chlorophyta or Bacillariophyta, which may explain the previous observation. In the north-west European algae strategic initiatives, the bioenergy (e.g., biodiesel) market is the most mentioned. The energy demand is growing worldwide, especially in the rapidly developing countries such as China and India [12]. Biofuels currently account only 1.9% of global transport fuel consumption and are projected to achieve a threefold increase over the next 20 years [13]. Brazil’s ethanol, derived from agricultural crops, is the most price-competitive biofuel in the world, which reflects the large investment of governmental agencies in research and technology that allowed the improvement of production processes, which in turn lowered biofuel manufacturing costs [14]. However, the food or fuel controversy derived from using agricultural crops for biofuel prompt the search for alternative sources as microalgae. Although these microorganisms have several advantages such as that they have a rapid growth rate, an ability to readily adapt to a wide range of climatic conditions, and that they do not compete for arable lands, the high initial capital investment and high biofuel production costs makes its exploitation for bioenergy purposes not feasible [15]. According to Moshood et al. [13], an economically efficient policy assistance may be required to overcome the main challenges found for microalgal biofuel production.

Ongoing efforts have been performed to reduce microalgal production costs; these include the evaluation of reactor design approaches, the development of biorefinery approaches, and the use of low-cost inputs for microalgal production. The use of wastewater for microalgal cultivation can offset the cost of freshwater and nutrients delivering environmental benefits such as the recycling of resources and reducing the nutrient discharges responsible for eutrophication in water bodies [16,17]. Numerous studies have evaluated the feasibility of microalgae cultivation for lipid production using wastewater from different sources: municipal [18,19,20], industrial [21], mining activity [22], landfill leachate [23], agricultural [24], and fish farm effluents [25]. Nevertheless, further research is needed to solve the drawbacks of the simultaneous microalgal biomass/product production and wastewater treatment, namely the everchanging chemical content of wastewater; the fact that not all metal ions and contaminants can be reduced using microalgae; and the need of wastewater pre-treatment (e.g., anaerobic digestate) to reduce organic compounds concentration, water turbidity, and low transparency, which affect microalgal growth [21,26].

According to the International Energy Agency [27], global energy-related carbon dioxide (CO_2_) emissions remained at 31.5 Gt, despite the decline in 2020. CO_2_ contributes up to 68% of the total greenhouse gases, the accumulation of which in the atmosphere has been considered as the main driver of climate changes [28]. Presenting higher carbon dioxide fixation efficiencies than terrestrial plants, microalgae have a pivotal role to play in future low-carbon economy [28,29]. In the literature, it is estimated that producing 280 t of microalgal dry biomass per ha per year using 9% of the incoming solar energy fixes roughly 513 t of CO_2_ [30]. In this sense, microalgae are often called “sustainable biofactories” due to their dual potential to mitigate/bioremediate atmospheric CO_2_ and produce a wide array of high-value compounds, which can be further enhanced through induced changes in its growth conditions.

Current prospects of algal biotechnology are turning their focus to high-value lipids production, namely polyunsaturated fatty acids (PUFA), which can be used in dietary supplements, functional food, pharmaceutical, and infant formula segments [31]. Some companies already produce ω3-PUFA with microalgal origin as dietary supplements or food ingredients (Oceans Alive (USA), Blue Biotech (Germany), Flora Health (USA)—*Nannochloropsis*; InnovalG (France)—*Odontella*) [32]. The global market size for ω3-PUFA in 2020 was estimated at US $16.2 billion, and its 2027 value projections are expected to reach US $36.9 billion at a compound annual growth rate (CAGR) of 12.5% over the forecast period of 2020–2027 [33].

Within Chlorophyta phylum, it is possible to observe that most algae farmers have been focusing on the production of oleaginous microalgae species, namely from *Chlorella*, *Tetraselmis*, *Botryococcus*, *Scenedesmus* genera, which are more specialized for biofuel production [7]. *Chlorella* sp., *Haematococcus pluvialis*, *Tetraselmis* sp., and *Chlorella vulgaris* are on the first line of microalgal species produced by algae farmers (Figure 2c). These microalgae have in common its versatility which allows to apply them in food, feed, energy production, and as a source of high-value molecules, which may reflect the efforts of algal farmers to target more than one market [34,35,36]. Furthermore, species belonging to *Haematococcus* and *Dunaliella* genera are mainly used for pigments exploitation, namely for astaxanthin and *β*-carotene production, respectively [7]. Protein is on the first line of development in human nutrition and health sectors, followed by pigments and lipids [7]. Accounting for the large number of oleaginous species already produced by algae farmers and the potentialities of microalgal lipids for high-value market, this review outlines the potential of microalgal high-value lipids for dietary supplements, cosmetics, and pharmaceutics, along with its health-promoting activities and optimization strategies.

## 2. Species Selection and Exploitation

Microalgae present a rich biodiversity comprising 40,000–50,000 described species with an estimation of nearly 800,000 existing species [37]. Chlorophyta comprises 6952 algal species [38], and Bacillariophyta is the most diversified group within microalgae, with more than 10,000 diatom species being described [39]. Despite this great diversity, only a few microalgal species have been exploited for biotechnological applications, with only 18 species of Chlorophyta and 10 species of Baccilariophyta phyla being produced by European algae farmers (Figure 2b). A smaller number of species is recorded for the other phyla: Cyanobacteria, Ocrophyta, Haptophyta, Rhodophyta, Euglenozoa, Charophyta, Miozoa, and Prasinodermatophyta.

The industrial production of microalgae heavily depends on biomass productivity, which is the most significant factor that can reduce the production cost levels [40]. To increase microalgae productivity, several strategies have been developed, namely the exploitation of the cultivation conditions to direct the metabolism towards desired product accumulation, selection and breeding of strains with increased biomass productivity, and genetic modification [40].

The selection of the right microalga considering specific culture conditions and desired product content could be performed through an exhaustive screen of scientific data through databases and literature, followed by physiological data collection (Figure 1). With respect to the product synthesized by microalgae, they can be applied in several fields from bioenergy (low-value market) to cosmetics and pharmaceutics (high-value market).

When exploiting the great biodiversity of microalgae as new natural sources of high-value phytochemicals, a screening pipeline should be developed including the following key aspects: *(i) growth*—for the successful improvement and progression of microalgae-based industries, the discovery and improvement of new fast-growing strains is essential [41]. Thus, the study of the maximum growth rate, maximum cell density, and dry cell biomass at different growth conditions, along with their amenability for heterotrophic/mixotrophic growth, are critical features to predict the feasibility of microalgae for large-scale production [42]; *(ii) robustness*—the selected microalgae should withstand variable local climatic conditions and be resistant to possible infections (e.g., other algae strains, grazers, bacteria, or viruses) in order to prevent large-scale crashes [43]. Nevertheless, strategies have been developed to combat and prevent contamination in microalgae cultivation such as the use of extreme conditions to create an unfavorable environment for the competitive organisms of the microalgae [44,45], and the use of allelopathic approaches to control microalgae cultivation [46]; *(iii) metabolite production*—in the production of microalgae for food and health purposes, the potential toxicity (phycotoxins) of some species as well as desired product (high-value products) accumulation should be considered.

### 2.1. Phycotoxins

Phycotoxins are causative agents of seafood-borne poisoning syndromes (e.g., ciguatera fish poisoning) in humans [47]. From the wide microalgal diversity, only around 200 species are health-threatening, with the main toxic microalgae belonging to dinoflagellates and diatoms groups [48]. Moreover, contamination of microalgae-based products resultant from unsuitable location of cultivation ponds (e.g., inflows of effluents containing pollutants) is a concern for human health [49]. Therefore, standard guidelines provided by international regulatory organizations, such as the Food and Drug Administration (United States of America) and European Food Safety Authority (Europe), guarantee that microalgae-based industries operate in conformity with safety requirements [48]. Through Table S1, is possible to have a brief insight on the regulations and directives applied in the European Union.

### 2.2. High-Value Products

Microalgae can be produced targeting different fields of application such as food, feed, pharmaceutical, personal care, and biomedicine (Figure 1). This versatility is derived from its ability to synthesize a multiplicity of metabolites distributed among pigments, vitamins, carbohydrates, lipids, and proteins/enzymes. Moreover, several biological properties attributed to microalgae, namely anti-inflammatory [50,51], anti-pyretic [51], and anti-cancer [52] activities, have shown its potential to high-value compound production.

**Figure 4:**
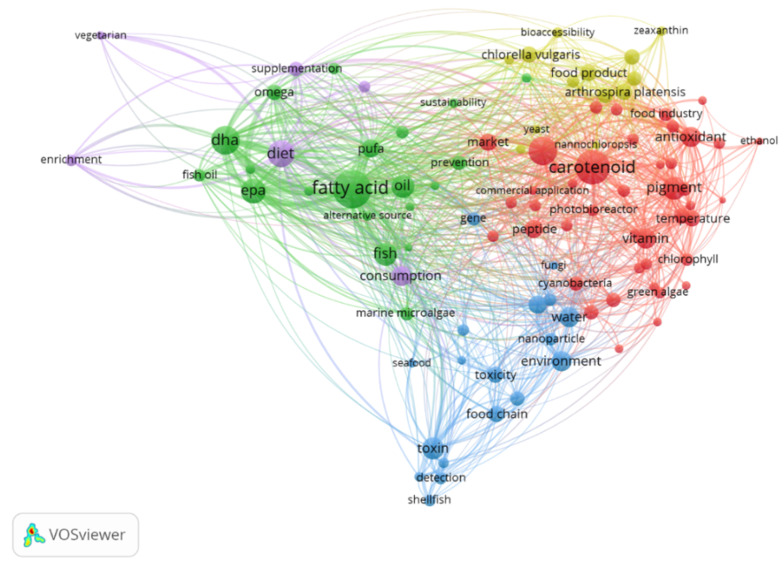
Concept’s network obtained with VOSviewer software [54] for the research on “microalgae AND food AND health” in Web of Science database [10].

Using the Cosmetic Ingredient Database (CosIng) [53], it is possible to see that several substances with microalgal origin have already been authorized, namely the oils from *Odontella aurita*, *Nannochloropsis oceanica*, *Chlorella minutisssima*, *Chlorella protothecoides*, and *Haematococcus pluvialis*. These can be incorporated into cosmeceuticals functioning as skin-conditioning emollient, skin protection, and anti-oxidants in cosmetic products [53]. Moreover, when analyzing research activities performed on microalgal production for food and health purposes, two main groups of metabolites stand out: fatty acids and carotenoids (Figure 4). These are mainly related to the lipid-soluble fraction of microalgae.

## 3. Polyunsaturated Fatty Acids (PUFA) Exploitation from Autotrophic Microalgae

PUFA are broadly known for their vital functions in human organisms [55]. For instance, docosahexaenoic acid (DHA, C22ω3) is enriched in human milk, plasma, and sperm, and along with arachidonic acid (AA, C20:4ω6) is concentrated in the membrane lipids of gray matter and in the visual elements of retina [56]. There is a lot of evidence that an adequate supply of these fatty acids improves visual acuity and infant cognitive development [56]. Moreover, several therapeutic properties have been attributed to the consumption of these fatty acids such as reduced risk of arthritis and cardiovascular diseases [57]. In contrast to microalgae, mammals do not have the ability to convert oleic acid (C18:1ω9) to the precursors of long-chain polyunsaturated fatty acids (LC-PUFA) biosynthesis pathway, and they poorly synthesize C20-C22 PUFA from dietary linoleic acid (LA, C18:2ω6) and *α*-linolenic acid (ALA, C18:3ω3).

### 3.1. PUFA—Synthesis by Microalgae

**Figure 5:**
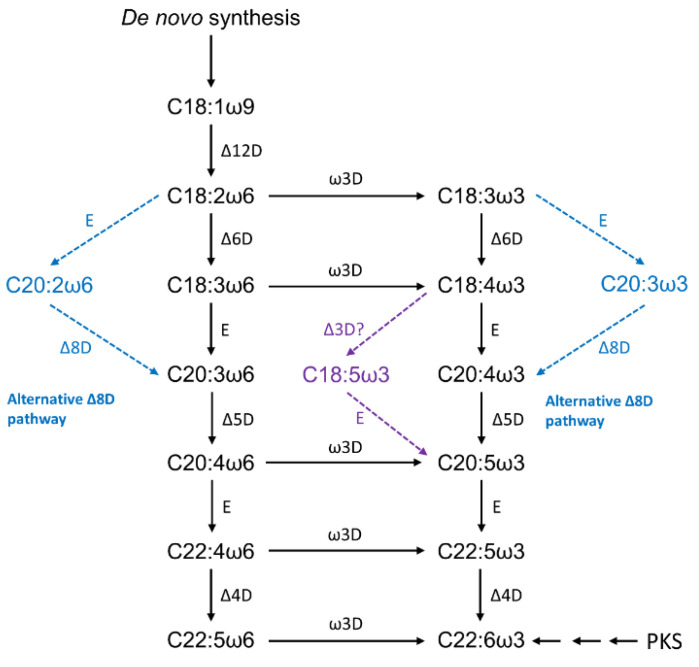
Biosynthesis of long-chain polyunsaturated fatty acids in microalgae [55,58,60]. ΔxD—“front-end” desaturase, adds a double bond at position x from carboxyl end; ωyD—“methyl-end” desaturase, adds a double bond at position y from methyl end; E—elongase, which catalyzes carbon chain extension; PKS—polyketide synthase.

LC-PUFA biosynthesis pathways by microalgae are initiated by Δ12 desaturation of C18:1ω9, producing LA, which might be further desaturated by a ω3-desaturase generating ALA (see Figure 5) [58]. The ω3-pathway is initiated with the Δ6 desaturation of LA and leads to the synthesis of ω3-LC-PUFA eicosapentaenoic acid (EPA, C20:5ω3) and DHA, whereas the ω6-pathway initiates with the Δ6 desaturation of ALA and produces the ω6-LC-PUFA, AA [58]. However, some EPA-producing eustigmatophytes, such as *Nannochloropsis* sp. and *Monodus subterraneus*, are thought to preferentially synthesize EPA via the ω6-pathway by the action of a ω3-desaturase, which catalyzes the conversion of AA to EPA [58,59].

As with other organisms, the microalgal fatty acid composition is known to vary among the different phylogenetic groups [58]. In Figure 5, is possible to visualize an alternative route for LC-PUFA biosynthesis: Δ8 desaturase pathway. According to Khozin-Goldberg [55], this pathway is known to exist in some microalgae, namely in haptophytes *Isochrysis galbana, Pavlova salina*, and *Emiliana huxleyi*, and the Euglenophyte *Euglena gracilis*. For the DHA-producing haptophyta *Isochrysis galbana*, a gene encoding a C22-Δ4 polyunsaturated fatty acid specific desaturase has been isolated and characterized [61]. The Trebouxiophyceae *Lobosphaera incisa* is a rare case in which AA is the major product of LC-PUFA biosynthesis in microalgae [55].

### 3.2. PUFA Role in Human Health

**Figure 6:**
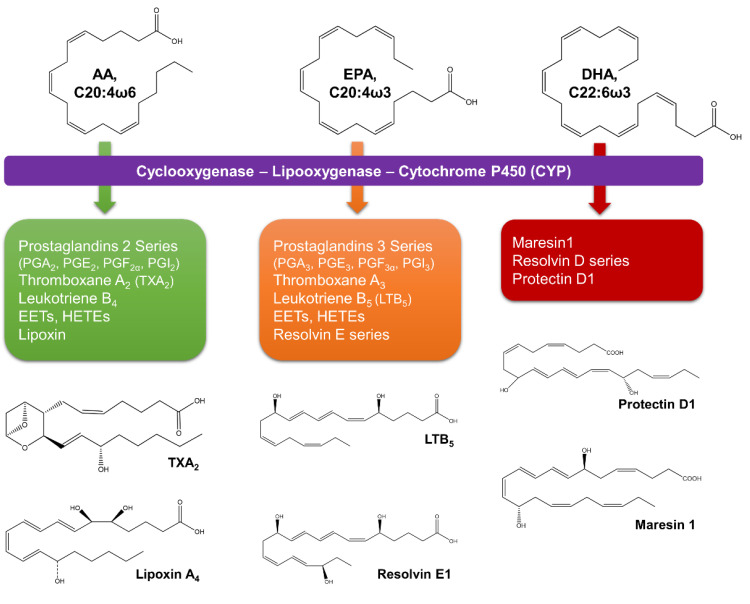
Lipid mediators of inflammatory process derived from arachidonic acid (AA), eicosapentaenoic acid (EPA), and docosahexaenoic acid (DHA) [63,64]. EETs—epoxyeicosatrienoic acids; HETEs—hydroxyeicosatetraenoic acids; PG—prostaglandin.

From microalgal lipids, PUFA are the most studied for their pharmacological potential. In human health, C20-C22 PUFA play important roles in many physiological and pathological processes [57]. Moreover, most of PUFA health-benefits are due to their key roles as lipid mediators in inflammatory processes and as important compounds for growth and development. EPA and DHA are parent compounds of specialized pro-resolving lipid mediators (protectins, resolvins, and maresins) that act as inflammatory brakes and promote the return of the affected site to homeostasis (Figure 6) [62,63]. As with ω3-PUFA, AA-derived lipoxins and their carbon-15 position epimers have beneficial effects on inflammation and resolution [62].

EPA competitively inhibits the utilization of AA by cyclooxygenasse/lipoxygenases to less pro-inflammatory mediators [65]. In contrast with eicosanoid products from AA (prostaglandin E2, thromboxane A2, and leukotriene B4), EPA-derived eicosanoids (thromboxane A3, and leukotriene B5) are weak inducers of inflammation and have attenuated platelet-aggregating and vasoconstriction abilities [65]. In mammalian cells, ω6- and ω3-fatty acids are not interconvertible because they lack ω3-desaturase; therefore, their balance in the diet is important [65]. In the Western diet, the greatest amounts of ω6-fatty acids (ω6:ω3 of 20:1) drive higher levels of AA eicosanoids products (e.g., prostaglandins, thromboxanes, leukotrienes, and hydroxy fatty acids), causing an imbalance between pro- and anti-inflammatory molecules and shifting the physiological state to one that is proinflammatory, prothrombotic, and proaggregatory [65]. Thus, a balanced intake of ω6- and ω3-fatty acids is crucial.

### 3.3. Microalgae—PUFA Enhancement Strategies

Although the largest share of the EPA/DHA oil market comes from wild fish, the declining fish stocks and susceptibility to contamination by pollutants (such as mercury) have turned the attention of PUFA exploitation to microalgae [66]. In this sense, the oxidative stability, sustainability, suitability for vegetarians, and the absence of fishy taste/smell are some of the advantages that make microalgae a feasible source for PUFA commercialization [66]. However, the immature production process is one of the weaknesses that must be surpassed for making PUFA exploitation from microalgae a feasible process [66].

New competitors of PUFA exploitation from microalgae are DHA producers belonging to thraustochytrids (*Traustochytrium* sp. and *Aurantiochytrium*) [66]. These unicellular heterothrophic organisms have already been authorized for food/feed/nutraceuticals and are marketed by DSM/Evonik and Source-Omega companies [66]. However, some disadvantages from the exploitation of these organisms for ω3-production (mostly produce DHA; the production chain increases pressure on arable land—since heterotrophic organisms need a sugar input to grow, and produces CO_2_) are opportunities for the production of ω3-fatty acids from microalgae [66].

Since the microalgae composition is known to vary with growth conditions, the study of strategies for microalgae PUFA enhancement is crucial to overcome the challenge of the undeveloped production process of phototrophic PUFA exploitation from microalgae. Although several strategies have been proposed to enhance microalgae lipids production, most of these have been projected for biofuel production. However, nowadays, more attention has been given towards PUFA production. Conventional approaches for enhancing microalgae lipid accumulation include nutrient stress (e.g., alterations in nitrogen, phosphorus, and carbon supply) or changes in cultivation conditions (e.g., light, and temperature) [67]. The main advantage of nutrient stress is its easy applicability at both lab and large-scale cultivation, while cultivation conditions such as light have high operational costs and are not easy to control within open cultivation systems [67]. Within nutrient regime alterations, nitrate limitation is a commonly employed strategy to enhance microalgal lipids quantity [67].

As essential constituent of proteins, nucleotides, vitamins, and coenzymes, any changes in the nitrogen source and concentrations can trigger growth changes and biochemical remodeling in microalgae species [68,69,70]. Although the study of Huang et al. [71] had the purpose of improving lipids properties of the microalgal strains *Tetraselmis subcordiformis* SHOU-S05, *Nannochloropsis oculata* SHOU-S14, and *Pavlova viridis* SHOU-S16, the insights on the fatty acid composition at different nitrogen concentrations enables to see some trends with respect to PUFA accumulation. Therefore, towards high nitrogen supplementations, the lipid content decreased, whereas the PUFA proportion increased for *N. oculata* and *P. viridis*. For *T. subcordiformis*, the highest PUFA percentage was registered at lower nitrogen supplementations as with the highest lipid content. As with Sukenik [72], this seems to suggest that the growth conditions for the maximization of PUFA production are similar to the conditions required to maximize biomass production for *N. oculata* and *P. viridis*.

Other cultivation conditions affecting microalgal growth and chemical diversity are the salinity, light intensity, and photoperiod. Mitra et al. [73] studied the effects of these factors in *Nannochloropsis gaditana* CCNM1032 strain, and concluded that the most positive factor for fatty acid enhancement was the photoperiod. In this study, maximal EPA productivities were achieved at 60 µmol photons m^−2^ s^−1^ and at a photoperiod regime of 18 h:6 h (light:dark); this observation was made for 1L cultures.

Two-stage cultivation and combined nutrient and abiotic stresses are novel approaches used to enhance the microalgal biochemical composition. In the two-stage cultivation, microalgae are first grown to gain higher biomass and are then exposed to different cultivation conditions to trigger the accumulation of desired product content. For *Nannochloropsis gaditana* IMTE1, a two-stage cultivation was studied by Xiao et al. [74]. This microalgal strain was firstly grown in batch culture for 6 days, washed, and then transferred for a chemostat culture with a fixed dilution rate and adjusted nitrate concentrations. In these cultivation conditions, the highest biomass (897.10 mg dw L^−1^; dw—dry weight) and EPA content (2.62% dw) were obtained at high nitrogen supplementations.

When combining nutrient and abiotic stresses, it is important to know the importance of each factor for the desired product accumulation, as well as its synergistic effects [67]. With the purpose of optimizing ω3-fatty acid production by *Pavlova lutheri*, Carvalho and Malcata [75] studied the combined effects of the dilution rate, light intensity, and CO_2_ concentration under continuous mode. The optimum conditions for EPA and DHA production were found in cultures supplied with 0.5% CO_2_, at a dilution rate of 0.297 d^−1^ and a light intensity of 120 µE m^−2^ s^−1^ [75]. Other combined nutrient and abiotic stresses are summarized in Table 1 for the following strains: *Chlamydomonas reinharditii* CC124, *Nannochloropsis gaditana* CCNM1032, *Phaeodactylum tricornutum* CS-29C, and *Chaetoceros muelleri* CS-176. The major disadvantage of the enounced novel approaches is that large-scale trials are required.

## 4. Sterols as an Underexploited Lipid Resource from Microalgae

When linking microalgae with food and human health, the predominant terms are fatty acids and carotenoids (Figure 4). Nevertheless, some microalgae are high-level producers of phytosterols, which have been playing a key role in the functional food market [79,80]. In addition, mixtures of phytosterols can function as skin conditioning in cosmetic products (creams and lipstick), and in pharmaceutics, they are gaining interest for the production of therapeutic steroids [53,81].

Plant-derived phytosterols have been added to food products for their ability to reduce serum cholesterol levels and prevent coronary heart diseases [79,82]. Cyanophya *Nostoc commune* var. *sphaeroides* lipid extracts have been found to inhibit the expression of key regulatory genes involved in cholesterol and fatty acid biosynthetic pathways; this property could contribute to lower serum cholesterol as well as triglyceride concentrations [83]. Phytosterols occur in four common forms: as free sterols (FS), as fatty acid esters (sterol is esterified to fatty acid; SE), as steryl glycosides (bound to sugar with a glycosidic bond; SG), and as acylated steryl glycosides (sugar moiety is acylated with a fatty acid; ASG) [84]. Due to the poor solubility of free phytosterols, major phytosterol/phytostanol products being marketed are in their conjugated forms (SE, SG, and ASG) as it is easier to add them into food products [84]. Table 2 summarizes information with respect to some brands that commercialize phytosterol fortified food products (yoghurts, spreads, soft cheese, and drinks), and other phytosterol-based products (supplements and paste). Although the raw materials used for phytosterols isolation for the food industry are tall oil (fat-soluble by-product obtained from trees) and vegetable oil, the scarcity of land resources has pulled the attention of scientific and industrial communities towards the search for new sustainable natural sources of phytosterols [85,86]. The concentration of phytosterols in different vegetable oils was estimated by Yang et al. [87] and ranged between 142.64 (camellia oil) and 1891.82 (rice bran oil) mg 100 g^−1^.

The potential of Chlorophytes *Dunaliella tertiolecta* and *Dunaliella salina* as sources of phytosterols was studied by Francavilla et al. [94]; in its study, the highest yields of total sterols were 1.3% and 0.89% dw for *D. tertiolecta* and *D. salina*, respectively. Another promising microalgal strain for phytosterol production, investigated by Ahmed et al. [79], was *Pavlova lutheri*, with the phytosterol content reaching up to 5.1% dw. Comparing these values with the ones previously mentioned for vegetable oils, it is possible to recognize that microalgae have the potential to become a useful alternative source of phytosterols to use as functional ingredients. Microalgal-derived phytosterols are mainly distributed among four groups: 4-desmethyl-Δ5-sterols; 4-desmethyl-Δ7-sterols; 4-methylsterols; and di-hydroxylated sterols [80], with 4-desmethyl-Δ5-sterols being the predominant phytosterol in microalgae [80,95]. According to Moreau et al. [84], 4-desmethyl sterols and stanols have been shown to inhibit the uptake of cholesterol from the intestine, resulting in a decrease of serum cholesterol levels. This once again strengthens the potential of using microalgae-phytosterols as a novel industrial application.

### 4.1. Sterol Synthesis by Microalgae

**Figure 7:**
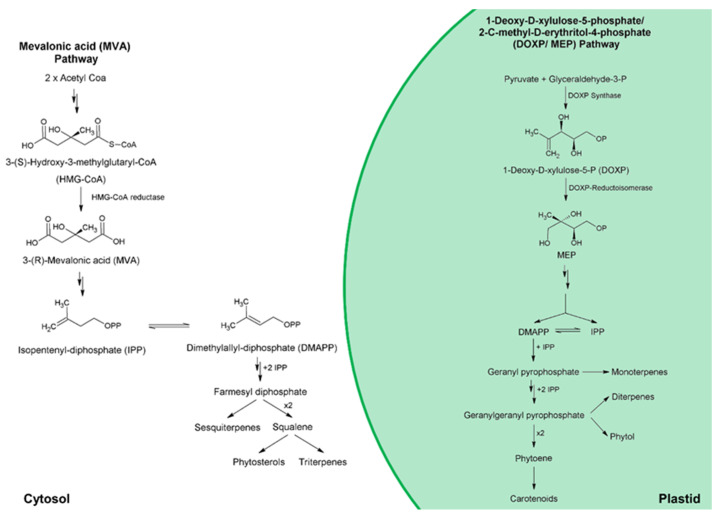
Generalized sterol biosynthesis pathway for microalgae [96,97].

For microalgae, there are two distinct and compartmentalized pathways for isoprenoid synthesis; these are (i) the mevalonic acid (MVA) pathway, in the cytosol; and (ii) the 1-deoxy-D-xylulose-5-phosphate/2-C-methyl-D-erythritol-4-phosphate (DOXP/MEP) pathway, in the plastid [80,96]. In general, microalgae possess both DOXP/MEP and MVA pathways [96]. Exceptions include Cyanophytes, Chlorophytes, as well as the Bacillariophyta *Haslea ostrearia*, and the Rhodophyta *Cyanidioschyzon merolae*, which produce sterols only from the MEP/DOXP pathway [97,98]. Figure 7 shows a generalized overview of sterol biosynthesis pathway for microalgae. Therefore, sterol biosynthetic pathway can be split into three main stages: (i) biosynthesis of isoprenoid precursors—isopentenyl pyrophosphate (IPP) and its isomer dimethylallyl pyrophosphate (DMAPP); (ii) biosynthesis of polyprenyl pyrophosphates such as farnesyl pyrophosphate (FPP); and (iii) squalene (precursor of all phytosterols) formation—dimerization of FPP [97]. According to Sasso et al. [97], there is evidence for the transport of IPP and polyprenyl pyrophosphates, such as FPP and geranyl pyrophosphate (GPP), across plastid membranes. Therefore, in algae such as Chlorophytes, in which sterols are only produced through MEP/DOXP pathway, IPP synthesized in the plastids could be exported to cytosol for the formation of sterols [99].

### 4.2. Microalgae-Derived Phytosterols Biological Activities

Phytosterols synthesized by microalgae have shown interesting biological activities including neuroprotective, anti-inflammatory, anti-cancer, neuromodulatory, immunomodulatory, and apoptosis inductive effects. From Table S2, it is possible to visualize that the predominant biological activity studied for microalgal-derived phytosterols was anti-inflammatory. The anti-inflammatory potential of phytosterols can be assessed in vitro through determination of nitric oxide (NO), prostaglandins (PG) and cytokines production, and/or expression of nitric oxide synthase (iNOS), and cyclooxygenase (COX-2), after cell treatment with inflammation stimulation agents such as concanavalin A (Con A) and lipopolysaccharide (LPS) [100,101,102,103,104]. Promising results were obtained for a sterol rich fraction of *N. oculata* which was found to inhibit NO production, and down-regulate LPS-stimulated protein levels of inducible iNOS and COX-2 in a dose-dependent manner [102]. Moreover, a study performed for *D. tertiolecta*, testing several mixtures of phytosterols, showed that ergosterol and a mix of ergosterol and 7-dehydroporiferasterol suppressed a highly pro-inflammatory cytokine (tumor necrosis factor alpha; TNF-α), a pleiotropic cytokine (interleukin (IL)-6), and increased the levels of an anti-inflammatory cytokine (IL-10), showing the anti-inflammatory potential of both sterols and suggesting that phytosterol anti-inflammatory properties might depend on the existence of a synergistic effect of these molecules [101].

Besides anti-inflammatory activity, the ability of phytosterols to cross the blood–brain barrier and act as acetylcholinesterase enzymes inhibitors has sparked the attention of neurodegenerative diseases research [105]. Fagundes et al. [105] studied the neuroprotective potential of *Phormidium autumnale* phytosterol-rich extracts and determined the anti-cholinergic, antioxidant, and anti-inflammatory capacities. In this study, the phytosterol-rich extract demonstrated higher in vitro neuroprotective activity than non-enriched extract, exhibiting a moderate–high anticholinergic potential, and showing to be an effective lipoxygenase inhibitor. This was further supported through molecular docking simulation, which showed the specificity of stigmasterol interaction with acetylcholinesterase active sites. Moreover, a previous study has found in vivo neuromodulatory activity of *D. tertiolecta*—derived phytosterols in selective brain areas of rats [85].

Through Table S2, it is possible to visualize that the biological studies with respect to phytosterols were mainly performed in vitro presenting some limitations, namely, these types of studies only give partial information on bio-functionality, and a lack of systemic factors [106]. However, they provide fast and inexpensive screening of bioactivities, have high sensitivity, and are easy to perform, manage, and interpret [106]. Moreover, it is crucial to highlight that most of the studies summarized in Table S2 are mainly performed in sterol-rich fractions and that future research targeting the potential of the different types of microalgae-derived phytosterols, including their functional activity and synergistic effects, is crucial for gaining in-depth knowledge of microalgae sterols potential.

### 4.3. Strategies for Sterol Enhancement

To boost phytosterol accumulation in microalgae, it is crucial to understand its trigger mechanisms. Table 3 summarizes some strategies already employed for inducing microalgae phytosterol accumulation. For Haptophyta *P. lutheri*, the effects of nutrient-induced changes, salinity, ultraviolet-C (UV-C) radiation, and sampling days have been assessed aiming phytosterol production [79,82]. From these variables, the most effective were UV-C radiation and sampling days [79,82]. Although the UV-C radiation equipment is simple and easy for operation and maintenance, this physical stressor has a great disadvantage for large-scale production, which is the significant cell damage connected with UV-C radiation mutagenic factor (as it attacks an organism’s deoxyribonucleic acid (DNA)) [107,108,109]. The mutagenic factor also poses a concern since little is known about the stability of modified algal strains or whether they can potentially take any environmental risks [110].

According to Ahmed et al. [79] and Ahmed and Schenck [82], *P. lutheri* did not increased its sterol content when subjected to peroxide hydrogen input, variations in nitrogen and phosphorus concentrations, and changes in salinity. Still, previous studies have described salinity-induced changes as an effective tool to induce phytosterol accumulation in microalgae. Francavilla et al. [94] studied the effect of different salinity concentrations for two Chlorophytas (*D. tertiolecta* and *D. salina*) using a different approach from the study performed to Haptophyta *P. lutheri*, and good yields of total sterols were observed at a lower salt concentration [94]. Thus, the different observations made in both studies might be derived from (i) species-specific differences and/or (ii) the different approaches used to impose salinity-induced changes.

Besides the growth conditions, the specific growth phase at which microalgal biomass is harvested can influence lipid yields and composition for specific purposes [70,114]. The effect of growth phase on the sterol content of dinoflagellate species (*Prorocentrum donghaiense*, *Prorocentrum minimum*, *Karenia mikimotoi*) in batch cultures was assessed by Chen et al. [112]. In this study, the sterol content of two microalgae was susceptible to changes in the growth phase (from exponential to stationary phase), with the greatest increase being verified for dinosterol (168%) in *P. minimum*, and for brassicasterol (423%) in *K. mikimotoi*. When assessing product accumulation over the growth phase, it is also important to take into consideration that there are some growth phases in which the production of stress-associated molecules (e.g., carotenoids, lipids) is still balanced with good amounts of growth-associated ones (e.g., proteins) [115]. This point is especially important for co-exploitation of high-value microalgae products.

Interactive effects of nutrient and abiotic factors have been reported to enhance the production of phytosterol in some freshwater microalgal species [111,116]. For instance, varying light intensities in both high- and low-phosphorus environments was shown to affect sterol accumulation for Chlorophyta *Scenedesmus quadricauda* and *Chlamydomonas globosa*, and Bacillariophyta *Cyclotella meneghiniana*, with sterol contents increasing with light intensity under high phosphorus [111]. Another strategy applied by Piepho et al. [116] was varying the temperature with phosphorus, as well as the silicate supply. For this study, *C. meneghiniana* increased its sterol content from low to high temperature, and this was even higher in the high-phosphorus treatment. Simultaneous effects of nutrient supply and abiotic factors in microalgae highlight the importance of investigating more than just one environmental factor when inducing the accumulation of a desired product for a field of application.

## 5. Carotenoids

**Figure 8:**
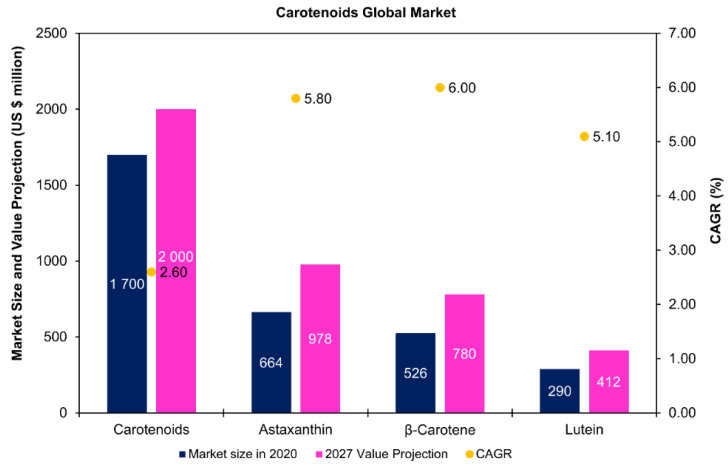
Carotenoids global market prospects—market size in 2020, 2027 value projection, and compound annual growth rate (CAGR). The values presented for carotenoids and lutein CAGR correspond to the forecast period of 2020–2027, while for astaxanthin and *β*-carotene, CAGR values correspond to the forecast period of 2021–2027. Data for carotenoids and lutein were collected from StrategyR [33], whereas data for astaxanthin and *β*-carotene were collected from Global Market Insights [124].

Some studies have explored the co-production of carotenoids with lipids [117] and fatty acids [118,119] in microalgae. Carotenoids are lipid-soluble compounds synthesized by microalgae and can be divided into carotenes (hydrocarbons) and xanthophylls (oxygenated hydrocarbons) [34,120]. As structural components of light-harvesting complexes, these compounds play key roles within microalgal cells, namely in the protection against excess irradiance, chlorophyll triplets, and reactive oxygen species [121]. Through Figure 8, it is possible to visualize that the global market for carotenoids was USD 1.7 billion in 2020. In microalgae, astaxanthin, *β*-carotene, and lutein are among the key carotenoids with high market potential [34,120,122]. Astaxanthin presents the highest value for the global market size, USD 663.89 million in 2020, and value projections for 2027, USD 977.74 million (Figure 7). *Haematococcus pluvialis* and *Dunaliella salina* are the most popular microalgal species exploited for the commercial production of astaxanthin and *β*-carotene, respectively [34]. In *Haematococcus*, astaxanthin is majorly esterified to palmitic acid (C16:0) and unsaturated fatty acids of the C18 family (C18:1, C18:2, and C18:3) [120,123].

Carotenoids are extensively used in food, feed, nutraceuticals, and cosmetics [125]. The consumption of a diet rich in carotenoids is often associated with positive effects on skin health, cancer, cardiovascular, neuronal, and gastrointestinal protection, and vision and immune system enhancement [125]. As with essential fatty acids and phytosterols, carotenoids cannot be synthesized by humans, which, in turn, must obtain these through their diet [125]. The beneficial effects of carotenoids to human health are thought to be derived from its potent anti-oxidant activity and the provitamin A activity of some carotenoids, which can be converted to retinal (e.g., *α*- and *β*- carotenes) [125].

Currently, astaxanthin is the best biological antioxidant, presenting a free-radical scavenging capacity 65 times more powerful than ascorbic acid (vitamin C) and 54 times stronger than *β*-carotene [126]. This carotenoid is best known for its use in aquaculture, namely for giving the pinkish-red color of salmonids, shrimps, lobsters, and crayfishes. In aquaculture this carotenoid is also used for its positive impacts on organisms’ immune-system and fertility [127]. Astaxanthin has a wide range of applications beyond aquaculture, namely in the food, cosmetic, and pharmaceutical industries [126]. Astaxanthin has several health-promoting properties, and it is used for anti-tumor therapies and prevention, treatment of neural damage interrelated with age-related macular degeneration, Alzheimer and Parkinson diseases [127].

Although natural astaxanthin has a notably higher antioxidant capacity and safety for human consumption, synthetically derived astaxanthin has a low production cost [128]. This makes astaxanthin from natural sources only account for less than 5% of the commercialized astaxanthin [128]. Given the metabolic plasticity of microalgae and its high growth rates, efforts have been displaced for reducing the costs of naturally derived astaxanthin. From astaxanthin producers, *H. pluvialis* is one of the richest sources of natural astaxanthin, accumulating up to 4% of astaxanthin on a dry weight basis. Table 4 summarizes different strategies already applied to *H. pluvialis* strains for prompting astaxanthin accumulation [128].

As can be seen in Table 4, most studies concerning astaxanthin accumulation by *H. pluvialis* have an induction stage from green- to red-phase. This distinction is associated with *H. pluvialius* cellular morphologies. Therefore, macrozooids (zoospores), microzooids, and palmella cellular morphologies are usually called “green-phase cells”, while hematocysts (aplanospores) are referred as “red-phase cells” [126]. The first predominate in favorable growth conditions, whereas the second occur under unfavorable environmental or culture conditions [126]. Since *H. pluvialis* accumulate large amounts of carotenoids, especially astaxanthin, under the red-phase, stress inputs such as nitrogen depletion and high light intensity are often applied to *H. pluvialis* to prompt astaxanthin production [126].

Several factors have already being studied for *H. pluvialis* in both green- and red-phases with the aim to prompt astaxanthin production, namely the effect of high light intensity exposure time, growth phase, cultivation mode, and the use of alternative substrates such as succinic acid, ethanol, and walnut shell extracts, as detailed in Table 4. Nonetheless, commercial production of astaxanthin from *H. pluvialis* still faces challenges such as the high operating costs of the mass cultivation of microalgae [128].

Besides *H. pluvialis* (5.8–22.7 mg g^−1^ dw), there are other astaxanthin-producing microalgae, such as *Scenedesmus vacuolatus* (1.5–2.7 mg g^−1^ dw), *Scotiellopsis oocystiformis* (6.4–10.9 mg g^−1^ dw), *Chlorella zofingiensis* (3.5–6.8 mg g^−1^ dw), *Neochloris wimmeri* (5.1–19.3 mg g^−1^ dw), and *Protosiphon botryoides* (12.9–14.3 mg g^−1^ dw).

The carotenoids database [134] at the access date of 8 October 2021 presented information on 1204 natural compounds distributed among 722 organisms from all domains of life. Xantophylls can be classified based on their chemical modifications as hydroxyl (lutein, zeaxanthin), epoxide (violaxanthin, neoxanthin), keto (astaxanthin), or carbonyl groups (canthaxanthin, capsanthin) [135]. Besides their health benefits and industrial applications, carotenoids can be useful biomarkers for microalgal distinction. Table 5 summarizes the main carotenoids and xantophylls that can be found for each algal phylum.

As can be seen in Figure 7, in microalgae, carotenoids are biosynthesized using the isoprenoid precursor’s IPP and DMAPP, which are further condensed to yield geranyl pyrophosphate. This molecule is elongated yielding geranylgeranyl pyrophosphate, which, in turn, can be dimerized to generate phytoene, the precursor of carotenoids [97]. Carotenoid accumulation often occurs when microalgal is exposed to some stress factors (e.g., nitrogen deficiency [137], ultraviolet-A radiation [118], salinity [138,139]) and growth is arrested [34]. Thus, for prompting carotenoids overproduction, for industrial scale, a two-stage cultivation strategy is often applied to first obtain higher biomass and then to trigger carotenoids accumulation [34].

## 6. Lipid Characterization

Carbon fixed by microalgae can be allocated into diverse metabolites. This distribution is dependent on microalgae metabolic and cellular organization, which in turn varies across distinct phylogenetic classes [140]. Thus, to improve microalgal biomass/product accumulation, several questions must be solved, namely how flux to desired product accumulation is controlled, and which interactions occur between and within metabolic pathways [141]. Metabolic profiling is a promising approach for identifying and quantifying the intracellular metabolic fluxes of microalgae, under different cultivation conditions [129].

Studies monitoring chemical diversity often screen a specific biological activity, a targeted compound class or individual molecule [142]. This constrains the development of microalgae-based industries once it overshadows the diversity of compounds produced by microalgae [143]. Several methods have been developed to allow a quantitative and simultaneous analysis of many groups of metabolites on complex mixtures by capillary electrophoresis mass spectroscopy (CE-MS), gas chromatography–mass spectroscopy (GC-MS), liquid chromatography–mass spectroscopy (LC-MS), nuclear magnetic resonance spectroscopy (NMR), and Fourier transform ion cyclotron resonance–mass spectroscopy (FTICR-MS) [144,145]. These strategies allow to save time, with respect to laborious isolation and quantification procedures, and give prompt information for complex mixtures, constituting a major advantage for microalgal-based industries [145].

Lipidomic comprises the detailed identification and quantification of lipid classes [146]. GC-MS has been widely used for the determination of the fatty acid compositions of microalgal lipids, which, in turn, are the basis of microalgae lipidomic studies [146]. However, the challenge with microalgal lipidomic studies lies in addressing its vast complexity and chemical heterogeneity [147]. GC-MS methods are recognized by their high detection sensitivity, accuracy, and excellent reproducibility; nevertheless, sample pretreatment (e.g., hydrolysis, derivatization) needs to be performed for samples [148]. A trimethylsilyl 2,2,2-trifluoro-N-trimethylsilylethanimidate (BSTFA) derivatization with GC-MS has been used as a simple, fast, low-cost, and powerful tool to gain in-depth knowledge on unknown but relevant lipids [149]. Through this non-target approach, it is possible to simultaneously analyze fatty acids, sterols, monoglycerides, aliphatic alcohols, glycosyl sterols, and other lipid-soluble molecules, such as *α*-tocopherols, without prior knowledge of the sample composition [150].

## 7. Conclusions

Until this stage of algal development, the production of bioenergy from microalgae is still not feasible. Thus, to take advantage of already exploited oleaginous microalgal species, algae farmers are turning their focus towards high-value lipids production for health and food sectors. Nevertheless, the great biodiversity of microalgae remains to be explored, holding back the opportunities that come from the wide diversity of compounds amongst microalgae taxa. Within lipids, the already recognized ω3-PUFA health-promoting properties are turning the focus of microalgal-based industries towards autotrophic ω3-PUFA production. However, more knowledge on the production strategies to prompt ω3-PUFA accumulation is lacking. Phytosterols are an unexplored lipid resource, which could pose an opportunity to explore them as food additives, as functional food, or dietary supplements. Lipid-soluble compounds carotenoids are the most extensively used in food, feed, nutraceuticals, and cosmetics. For high-value lipid exploitation, increasing the knowledge on new, simple, and cost-effective strategies to increase the production of these molecules is necessary. Additionally, the use of high-throughput methods that allow the identification and quantification of a wide array of lipid components are needed. The promising biological activities of microalgal-derived phytosterols show that the future of microalgal high-value lipids should not be restricted to fatty acids.

## Figures and Tables

**Table 1:** Some strategies used for microalgae lipid enhancement and its impact on polyunsaturated fatty acids accumulation.

Microalga	Strain	Factors Used	Biomass	Lipid	PUFA Content	Notes	Ref.
*Chlamydomonas reinhardtii*	CC124	Phosphorus supplementation under nitrogen deficiency			PUFA: 17.15–45.23 µg mg^−1^; DHA: 0.09–0.17 µg mg^−1^		[76]
*Chlamydomonas reinhardtii*	CC124	Acetate input (1, 2, and 4 g L^−1^ sodium acetate)	1.08–2.49 g L^−1^		PUFA: 28.84–51.58 µg mg^−1^; DHA: 0.03–0.09 µg mg^−1^		[76]
*Heterochlorella* *luteoviridis*	BE002	Temperature (22, 27 and 32 °C) and NaNO_3_ content (12, 24, 36, 48 or 60 mg L^−1^ of N-NO_3_)	0.48 g L^−1^ d^−1^	82.5–99.1 mg g^−1^	PUFA: 34.4–40.7% TFA	Biomass productivity obtained at higher nitrogen conditions	[77]
*Nannochloropsis oceanica*	IMET1	Nitrogen-deficiency stress (60, 120, and 2200 µmol L^−1^ NO_3_^2−^)	319.10–897.10 mg L^−1^	34.04–56.17% dw	EPA: 1.77–2.62% dw	The highest EPA amount was observed at 2200 µmol L^−1^ NO_3_^2−^, in contrast to the lipid content	[74]
*Tetraselmis* *subcordiformis*	SHOU-S05	Nitrogen supplementation (0, 0.22, 0.44, 0.88 and 1.76 mmol N·L^−1^)		13.40–29.77%	PUFA: 57.97–62.59% TFA EPA: 2.92–3.85% TFA	The highest values of PUFA and EPA were obtained at 0.22 mmol N L^−1^	[71]
*Nannochloropsis oculata*	SHOU-S14			22.5–35.85%	PUFA: 46.10–53.69% TFA EPA: 29.34–35.51% TFA	The highest values of PUFA and EPA were obtained at 1.76 mmol N L^−1^	[71]
*Pavlova viridis*	SHOU-S16			26.45–32.10%	PUFA: 26.94–41.28% TFA EPA: 9.52–15.71% TFA DHA: 2.39–7.17% TFA	The highest values of PUFA and EPA were obtained at 1.76 mmol N L^−1^	[71]
*Nannochloropsis gaditana*	CCNM1032	Salinity (20, 30, 35, and 40 g L^−1^), light intensity (60 and 150 μmol photons m^−2^ s^−1^), and photoperiod (24/0, 18/6, 12/12, 6/18 and 0/24 light/dark hour)	45.01 mg L^−1^ d^−1^	14.63 mg L^−1^ d^−1^	EPA: 19.13–37.83% TFA	Biomass and lipid productivities were obtained at a salinity gradient of 20 g L^−1^	[73]
*Phaeodactylum* *tricornutum*	CS-29C	Nitrogen source (nitrate, ammonium, and urea) and ultraviolet (UV) radiation (UV-A: 315–400 nm; UV-B: 280–315 nm)			PUFA: 34.89–48.85% TFA EPA: 18.86–23.42% TFA DHA: 1.49–2.52% TFA		[78]
*Chaetoceros muelleri*	CS-176				PUFA: 29.26–36.76% TFA EPA: 9.61–14.23% TFA DHA: 0.75–1.42% TFA		[78]
*Pavlova lutheri*	SMBA60	CO_2_ concentrations (0–2% *v*/*v*), light intensity (75 and 120 µmol photons m^−2^ s^−1^) and cultivation mode (batch and continuous)	0.900 g L^−1^	132.5 mg L^−1^	EPA: 3.61 mg L^−1^ d^−1^ DHA: 1.29 mg L^−1^ d^−1^	Values obtained at 0.5% (*v*/*v*) CO_2_, a dilution rate of 0.297 d^−1^, and a light intensity of 120 µmol photons m^−2^ s^−1^	[75]

PUFA—polyunsaturated fatty acids; DHA—docosahexaenoic acid; EPA—eicosapentaenoic acid; TFA—total fatty acids; dw—dry weight.

**Table 2:** Phytosterols marketed as low cholesterol agents.

Manufacturer	Brand	Products	Source	Ref.
Raisio group	Benecol	Soft cheese Yoghurt drinks Yoghurts Spreads	Plant phytostanol esters	[88]
Upfield	Flora ProActiv	Spreads Drinks	Plant sterols	[89]
Goodman Fielder	Logicol	Spread	Plant sterols	[90]
Archer Daniels Midland (ADM)	CardioAid	Powder Paste soluble in oils and fats	Plant sterols	[91]
Cargill	CoroWise	Dietary foods * Beverages Supplements	Plant sterols (phytosterols and steryl esters)	[92]
Lipofoods	Lipophytol	Water-dispersible powder	Plant sterols (from soy or pine tree origin)	[93]

* Foods with plant sterols and commercialized include emulsified sterols (ES200); fine particle sterols (FP100); granular phytosterols (FP300); steryl esters (SE-C100); water dispersible steryl esters (WDSE-33).

**Table 3:** Some strategies evaluated for microalgae sterol enhancement.

Microalga	Variables Studied	Total Sterols	Major Sterols	Observations	Ref.
*Diacronema lutheri (syn. Pavlova lutheri)*	UV-C radiation (50–250 mJ m^−2^) Hydrogen Peroxide (H_2_O_2_: 1–500 µM)	9.9–20.3 mg g^−1^ dw 19.5–30.9 mg g^−1^ dw	Poriferasterol Clionasterol 4-*α*-methylporiferast-22-enol Methylpavlovol Epicampesterol	↑TS was found at 100 mJ cm^−2^ No significant increase of TS due to H_2_O_2_	[82]
	Combined effects of sampling days (2, 4, 6, 12, 14, and 16), and salinity (15, 25, 35, and 45‰)	20.29–51.86 mg g^-−1^ dw		Significant differences were observed between sampling days but not for the different salinities	[79]
*Dunaliella salina*	Salinity (0.6, 1.4 and 2.1 M NaCl)	0.89% dw	7-Dehydroporiferasterol Ergosterol	Good yields of TS were found at lower salt concentrations (0.6 M)	[94]
*Dunaliella tertiolecta*		1.3% dw			
*Scenedesmus quadricauda*	Combined effects of light intensity (30, 60, 140, 230, and 490 µmol photons m^−2^ s^−1^), and phosphorus (1–50 µM)	8–13 µg mg C^−1^	Fungisterol Chondrillasterol 22-Dihydrochondrillasterol	In the high-P TS increased with light intensity	[111]
*Cryptomonas ovata*		7–8 µg mg C^−1^	Epibrassicasterol Stigmasterol	No significant changes in TS	
*Cycotella meneghiniana* SAG 1020-1a		5–8 µg mg C^−1^	24-Methylene-cholesterol 22-Dihydrobrassicasterol		
*Chlamydomonas globosa*		3–4 µg mg C^−1^	Ergosterol Fungisterol		
*Prorocentrum donghaiense*	Temperature (15, 20, and 25 °C) N:P supply (10:1, 24:1, and 63:1 molar ratios) Growth phase (exponential and stationary growth phases)	Brassicasterol: 0.03–0.12 pg cell^−1^ Dinosterol: 0.15–1.54 pg cell^−1^	Brassicasterol Dinosterol	Growth phase changes showed the most pronounced effects, while temperature and nutrient deficiency had moderate effects on sterol contents	[112]
*Prorocentrum minimum*		Brassicasterol: 0.04–0.20 pg cell^−1^ Dinosterol: 0.28–1.83 pg cell^−1^			
*Karenia mikimotoi*		Brassicasterol: 0.07–1.56 pg cell^−1^ Dinosterol: 0.20–1.30 pg cell^−1^			
*Thalassiosira pseudonana* CCMP1335	Rapid cooling (18 to 4 °C) Salinity (10, 17, 25, 30, 39, 47, 53 and 61‰)		24-Methylenecholesta-5,24(24’)-dien-3β-ol Fucosterol Isofucosterol Cholesterol		[113]
*Phaeodactylum tricornutum* CCMP632			24-Methylenechol esta-5,24(24’)-dienol Fucosterol Isofucosterol Cholesterol	Shifts its sterol content at a reduced temperature	
*Chaetoceros muelleri* CCMP1316			Brassicasterol Campesterol Cholesterol	Rapid cooling did not significantly change sterols relative abundance	

**Table 4:** Strategies applied for enhancing astaxanthin production for *Haematococcus pluvialis* strains.

		Induction Stage				
Strain	Medium	Green-Phase Cells	Red-Phase Cells	Factors Studied	Observations	Ref.
*H. pluvialis* Flotow 1844em. Wille K-0084	mBG-11	N-replete 5 days 75 μmol photons m^−2^ s^−1^	N-free 2 × 10^5^ cells ml^−1^ 350 μmol photons m^−2^ s^−1^ 48 or 96 h	High light intensity time exposure: 48 h—0, 6, 12, 24, 36, 48 h 96 h—0, 24, 48, 72, 96 h	Astaxanthin dominated carotenoid composition: 92.6% Car and 97.7% Car, after 24 and 48 h respectively	[129]
*H. pluvialis* IPPAS H-2018 (former BM1)	BG-11	N-replete 40 μmol photons m^−2^ s^−1^ 60 μmol photons m^−2^ s^−1^	N-free 480 μmol photons m^−2^ s^−1^	Green-phase cells: CO_2_ concentrations (5, 10, 20%); Growth phase (exponential and stationary). Red-phase cells: CO_2_ concentrations (5, 10, 20%)	5% CO_2_ resulted in a higher astaxanthin productivity.	[130]
*H. pluvialis* NIES 14	BG-11	N-replete	N-free	Organic carbon: 0.5% (*v*/*v*) of methanol, ethanol, glycol, acetaldehyde, isopropanol, and glycerol Ethanol treatment: 1, 2, 3 and 5% (*v*/*v*) Light Intensity: low (25 μmol photons m^−2^ s^−1^) and high (150 μmol photons m^−2^ s^−1^)	Astaxanthin productivity reached 11.26 mg L^−1^ d^−1^at 3% (*v*/*v*) ethanol	[131]
*H. pluvialis* LUGU (KM115647.1)	BG-11	N-replete 30 μmol photons m^−2^ s^−1^	N-free 250 μmol photons m^−2^ s^−1^	Cultivation mode (batch and batch-fed) Concentration of SA (0.25, 0.5, 1.0, 2.0 and 4.0 mM) on fed-batch operation (7, 9, and 11 days)	Maximum values of astaxanthin (35.88 mg g^−1^) and lipid (54.79%) contents were obtained after supplementation of SA on day 7	[132]
*H. pluvialis* NIES-144	NIES-C	50 μmol photons m^−2^ s^−1^	250 μmol photons m^−2^ s^−1^	Cultivation mode (batch and semi-continuous Large-scale cultivation	Induction stage lasted 8 and 20 days; For semi-continuous cultivation light intensity was constant (250 μmol photons m^−2^ s^−1^); Semi-continuous process produced 700.4 mg L^−1^ of astaxanthin over 60 days	[128]
*H. pluvialis LUGU*	BG-11	N-replete 30 μmol photons m^−2^ s^−1^	N-free 250 μmol photons m^−2^ s^−1^	Walnut shell extracts (WSE) concentrations (10, 15, and 20%)	The highest astaxanthin (29.53 mg g^−1^) and lipid (51.75%) occurred with 15% of WSE	[133]

Car—carotenoids; N—nitrogen; SA—succinic acid.

**Table 5:** Main carotenoids and xanthophylls of the algal phyla [136].

Phylum	Carotenoids	Xantophylls
Cyanobacteria	*β*-carotene	Myxoxanthin, zeaxanthin
Prochlorophyta	*β*-carotene	Zeaxanthin
Glaucophyta	*β*-carotene	Zeaxanthin
Rhodophyta	*α*- and *β*-carotene	Lutein
Cryptophyta	*α*-, *β*-, and *ε*-carotene	Alloxanthin
Ocrophyta	*α*-, *β*-, and *ε*-carotene	Fucoxanthin, violaxanthin
Haptophyta	*α*- and *β*-carotene	Fucoxanthin
Dinophyta	*β*-carotene	Peridinin, fucoxanthin, diadinoxanthin, dinoxanthin, gyroxanthin
Euglenophyta	*β*- and *Ƴ*- carotene	Diadinoxanthin
Chlorarachniophyta	absent	Lutein, neoxanthin, violaxanthin
Chlorophyta	absent	Lutein, prasinoxanthin

## Data Availability

The data presented in this study are available on request from the corresponding author.

## Supplementary Materials

The following are available online at https://www.mdpi.com/article/10.3390/md19100573/s1: Table S1. Insight on the legislation available in the European Union to regulate the food market, Table S2. Biological activities studied for microalgae-derived phytosterols in cell culture experiments and animal models.
