# Supplementary material for: Microalgae as Sustainable Biofactories to Produce High-Value Lipids: Biodiversity, Exploitation, and Biotechnological Applications
Source: Mar Drugs. 2021 Oct 14;19(10):573. doi: 10.3390/md19100573 (PMC8540142; doi:10.3390/md19100573)
Supplement: Supplementary file 1 [file marinedrugs-19-00573-s001.zip › marinedrugs-1413351-SI-re.pdf]

marine drugs

MDPI

Supplementary Material

Microalgae as Sustainable Biofactories to Produce High-Value Lipids: Biodiversity, Exploitation, and Biotechnological Applications

Tomásia Fernandes [1,2] and Nereida Cordeiro [1,2,*]

[1] Laboratory of Bioanalysis, Biomaterials, and Biotechnology (LB3), Faculty of Exact Sciences and Engineering, University of Madeira, Campus Universitário da Penteada, 9020-105 Funchal, Portugal; tomasia.fernandes@uma.pt
[2] Interdisciplinary Centre of Marine and Environmental Research (CIIMAR), University of Porto, 4450-208 Matosinhos, Portugal
* Correspondence: ncordeiro@staff.uma.pt

Table S1. Insight on the legislation available in European Union to regulate food market.

	Regulations and Directives	Brief description	Ref.
Food supplements	Directive 2002/46 EC	Sets-out rules on food supplements to protect consumers<br>Ensure that products are not provided with misleading information	[1]
New (novel) foods	Regulation (EU) 2015/2283	Rules for the placing of novel foods on the market in the EU	[2]
Feed additives	Regulation (EC) No 1831/2003	Standardized procedure for authorizing feed additives<br>Rules for labelling, placing on the market and use	[3]
Foods for specific groups	Regulation (EU) No 609/2013	Sets-out compositional and labelling requirements for infant formulae<br>Follow-on formulae intended for use by Community infants in good health	[4]
Nutrition and health claims on food	Regulation (EC) No 1924/2006	Ensure a high level of consumer protection<br>Give the consumer the necessary information to make choices<br>Create equal conditions of competition for the food industry	[5]
Medicinal products	Regulation (EC) No 726/2004	Guarantee high standards of quality and safety of medicines in the EU	[6]

Table S2. Biological activities studied for microalgae-derived phytosterols in cell culture experiments, and animal models.

Microalga	Extract description	Activity	Assay	Model	Ref.
Phormidium autumnale	Phytosterol-rich fraction	Neuroprotective	*In vitro*<br>*In silico*		[7]
Nannochloropsis oculata	Sterol rich fraction	Anti-inflammatory<br>Anti-cancer	*In vitro*	RAW 264.7 macrophage cells<br>HL-60, A-549, HEP-3B, HCT-116, and SW-480 cancer cells	[8]
Navicula incerta	Isolated stigmasterol	Anti-proliferative	*In vitro*	HepG2 cells	[9]
Dunaliella tertiolecta	Phytosterols<br>Mix of ergosterol, and 7-dehydroporiferasterol<br>Mix of acetylated ergosterol and 7-dehydroporiferasterolergosterol	Immunomodulatory<br>Anti-inflammatory	*In vitro*	Sheep peripheral blood mononuclear cells	[10]
	Phytosterols	Neuromodulatory	*In vivo*	Rats	[11]
Chlorella vulgaris	Isolated sterols	Anti-inflammatory	*In vivo*	ICR mice	[12]

References

1. Publications Office. Ensuring safe food supplements in the EU Available online: https://eur-lex.europa.eu/legal-content/EN/LSU/?uri=celex:32002L0046 (accessed on Apr 14, 2021).
2. Publications Office. New (novel) foods — rules from 2018 Available online: https://eur-lex.europa.eu/legal-content/en/LSU/?uri=CELEX:32015R2283 (accessed on May 24, 2021).
3. Publications Office. Use of additives in feedingstuffs Available online: https://eur-lex.europa.eu/legal-content/en/LSU/?uri=CELEX:32003R1831 (accessed on May 24, 2021).
4. Publications Office. Foods for specific groups Available online: https://eur-lex.europa.eu/legal-content/EN/LSU/?uri=celex:32013R0609 (accessed on May 24, 2021).
5. Publications Office. Nutrition and health claims made on foods Languages Available online: https://eur-lex.europa.eu/legal-content/en/LSU/?uri=CELEX:32006R1924 (accessed on May 24, 2021).
6. Publications Office. Safe medicines for Europeans — European Available online: https://eur-lex.europa.eu/legal-content/EN/LSU/?uri=celex:32004R0726 (accessed on May 24, 2021).
7. Fagundes, M.B.; Alvarez-Rivera, G.; Mendiola, J.A.; Bueno, M.; Sánchez-Martínez, J.D.; Wagner, R.; Jacob-Lopes, E.; Zepka, L.Q.; Ibañez, E.; Cifuentes, A. Phytosterol-rich compressed fluids extracts from Phormidium autumnale cyanobacteria with neuroprotective potential. *Algal Res.* **2021**, *55*, doi:10.1016/j.algal.2021.102264.
8. Sanjeewa, K.K.A.; Fernando, I.P.S.; Samarakoon, K.W.; Lakmal, H.H.C.; Kim, E.A.; Kwon, O.N.; Dilshara, M.G.; Lee, J.B.; Jeon, Y.J. Anti-inflammatory and anti-cancer activities of sterol rich fraction of cultured marine microalga nannochloropsis oculata. *Algae* **2016**, *31*, 277–287, doi:10.4490/algae.2016.31.6.29.
9. Kim, Y.S.; Li, X.F.; Kang, K.H.; Ryu, B.M.; Kim, S.K. Stigmasterol isolated from marine microalgae Navicula incerta induces apoptosis in human hepatoma HepG2 cells. *BMB Rep.* **2014**, *47*, 433–438, doi:10.5483/BMBRep.2014.47.8.153.
10. Caroprese, M.; Albenzio, M.; Ciliberti, M.G.; Francavilla, M.; Sevi, A. A mixture of phytosterols from Dunaliella tertiolecta affects proliferation of peripheral blood mononuclear cells and cytokine production in sheep. *Vet. Immunol. Immunopathol.* **2012**, *150*, 27–35, doi:10.1016/j.vetimm.2012.08.002.
11. Francavilla, M.; Colaianna, M.; Zotti, M.; G. Morgese, M.; Trotta, P.; Tucci, P.; Schiavone, S.; Cuomo, V.; Trabace, L. Extraction, Characterization and In Vivo Neuromodulatory Activity of Phytosterols from Microalga Dunaliella Tertiolecta. *Curr. Med. Chem.* **2012**, *19*, 3058–3067, doi:10.2174/092986712800672021.
12. Yasukawa, K.; Akihisa, T.; Kanno, H.; Kaminaga, T.; Izumida, M.; Sakoh, T.; Tamura, T.; Takido, M. Inhibitory effects of sterols isolated from Chlorella vulgaris on 12-0-tetradecanoylphorbol-13-acetate-induced inflammation and tumor promotion in mouse skin. *Biol. Pharm. Bull.* **1996**, *19*, 573–576.
